# Immune Monitoring Goes Viral – Torque Teno Virus for Immunologic Risk Stratification After Kidney Transplantation

**DOI:** 10.3389/ti.2025.15074

**Published:** 2025-11-14

**Authors:** Konstantin Doberer, Sebastian Kapps, Frederik Haupenthal, Gregor Bond

**Affiliations:** Division of Nephrology and Dialysis, Department of Medicine III, Medical University of Vienna, Vienna, Austria

**Keywords:** torque teno virus, immune monitoring, biomarker, kidney transplantation, risk stratification

## Abstract

Using biomarkers to tailor immunosuppressive therapy after kidney transplantation was proposed to improve clinical care. Timely and individual adaptions of immunosuppression could reduce therapy-related side effects, such as infections, cardiovascular morbidity and malignancy, and further lower the risk of allograft rejection. Despite promising preliminary studies, evidence for implementing such a biomarker in clinical care is insufficient. Prominent candidates for immunologic monitoring after kidney transplantation include donor human leukocyte antigen-specific antibodies, donor-derived cell-free DNA, urinary chemokines and peripheral transcriptomics. In addition, the quantification of Torque Teno virus, a highly prevalent and non-pathogenic virus that was shown to associate with outcomes linked to immunocompetence, has been proposed for immunologic monitoring. This review summarises the prospects and limitations of Torque Teno virus for immunologic risk stratification after kidney transplantation in the context of current state-of-the-art. It will focus on cut-off values of plasma Torque Teno virus load that might be useful to guide immunosuppression in the clinical care of kidney transplant recipients, and highlights recently proposed indications of Torque Teno virus-guided immunosuppression.

## Introduction to the Clinical Problem

Organ transplantation, which prolongs and improves the lives of patients with end-stage renal disease [1, 2], is complicated by the need to dampen the hosts’ immune response. Patients on life-long immunosuppressive drug regimens are at increased risk of infections, cancer and cardiovascular morbidity [3, 4]. In addition, even modern immunosuppressive regimens cannot entirely abrogate alloimmune processes [5, 6]. Therefore, biomarker-guided immunosuppression was proposed to reduce complications caused by extensive or insufficient immunosuppression. Many candidate markers have been studied in recent decades with some very promising results [7, 8]. However, their transition into clinical use is faltering, and scientific evidence for integrating immunologic monitoring markers into routine care remains insufficient. While there are many reasons, they nearly always come down to a lack of validation in interventional trials, potentially compounded by high costs and a lack of reimbursement. Currently, the most promising candidates for immunologic monitoring after kidney transplantation are donor-specific antibodies (DSAs), donor-derived cell-free DNA (dd-cfDNA), urinary chemokines and peripheral transcriptomics.

Unlike the abovementioned biomarkers, which mainly reflect organ damage or alloreactivity, the Torque Teno virus (TTV) – a highly prevalent and non-pathogenic virus of the family Anelloviridae – was shown to be associated with outcomes linked to both excessive and insufficient immunosuppression. Therefore, TTV may serve as a biomarker indicating the net state of the immune system and thus could complement existing injury and alloreactivity markers. A gradual deviation from an ‘optimal’ TTV load toward a high or low load was shown to correlate with an increased risk of infections and allograft rejection [9]. More recent data show associations with malignancy and the immune response after vaccination [10]. The introduction of a quantitative polymerase chain reaction (qPCR) assay has made TTV a readily measurable parameter. However, the framework for implementing it in routine clinical care requires further definition. One multicentre interventional trial assessing the clinical value of TTV-guided immunosuppression in kidney transplant recipients was successfully concluded in May 2025 (TTVguideIT, EUCT number: 2022-500024-30-00) [11], and another started recruiting in April 2025 (TAOIST, ClinicalTrails.gov ID: NCT06829719).

This article will reflect on the role of TTV as an immune marker in the clinical care of kidney transplant recipients, focusing on relevant data that have emerged over the last 3 years. Previous relevant studies will only be mentioned briefly, as they have been discussed in detail earlier [9]. The role of TTV from the clinical virologist’s perspective has been reviewed by others recently [12, 13]. Biomarkers other than TTV will only be discussed briefly, as current reviews cover peripheral blood gene expression, dd-cfDNA, urinary chemokines and other emerging candidates [8, 14, 15].

## Current Status of Immune Monitoring in Clinical Care of Kidney Transplant Recipients

Despite a long history of immune monitoring research in kidney transplantation, no biomarker is supported by sufficient evidence to enter clinical practice. Standardised assays, rigorous validation and interventional trials demonstrating the clinical value of biomarkers when added to the standard of care are prerequisites for broad implementation. Lately, some studies yielded promising results and a recent consensus statement of the European Society of Organ Transplantation cautiously discussed recommendations in favour of biomarker monitoring in certain settings while urgently calling for interventional trials [16]. However, the study design in this context is complicated. Endpoints commonly used in interventional clinical trials, such as allograft rejection or loss, have low incidences, necessitating the inclusion of high-risk patients or large populations. Consequently, a randomised controlled trial (RCT) assessing *de novo* DSA-triggered optimisation of immunosuppression suffered from lower-than-expected rejection and biopsy rates and failed to show improved allograft survival [17]. Similarly, a multicentre RCT evaluating urinary monitoring of C-X-C motif chemokine ligand 10 (CXCL10) in addition to the standard of care could not demonstrate a reduction in rejection rates during the first year post-transplantation, at least partly due to low biopsy and rejection rates [18].

Besides sufficient power, outcome selection is crucial in biomarker trial design. For example, while dd-cfDNA was developed as an injury marker, several studies focused on dd-cfDNA as a tool to rule out rejection and avoid invasive diagnostics. This demands close and longitudinal monitoring in large patient cohorts, which, even with a high negative predictive value, may easily become very resource-demanding. A more effective approach might be ruling in rejection in high-risk patients. Consistent with this approach, the first positive results came from a single-centre RCT demonstrating an accelerated diagnosis of late antibody-mediated rejection (ABMR) in patients with *de novo* DSA if dd-cfDNA was added to the standard of care [19]. Following these first promising results, validation in large and diverse multicentre cohorts is now eagerly awaited.

The nature of the biomarker should also be considered carefully in the study design and outcome selection phase. For example, injury markers might not be useful for predicting potential future adverse events [20]. Biomarkers reflecting the state of immunosuppression could overcome this problem. TTV is a promising candidate due to its link to both extensive and insufficient immunosuppressive burden before adverse events manifest. Quantifying immunocompetence using TTV may serves as a complement to markers of graft injury (e.g. dd-cfDNA) and alloreactivity (e.g. DSA).

Alternative approaches to quantify the net state of the immune system rely on cellular assays. One proposed assay was the ImmuKnow, which did not enter clinical practice despite promising results within a single centre RCT [21]. Recently, another RCT including paediatric patients showed promising data for Tvis [22], an assay that relies on quantifying virus-specific T cells. Because of the complex logistical procedures, most of the patients (86%) where randomised only at one study site. Multicentre validation will be crucial as the complexity of the assay might pose an obstacle to standardisation and implementation.

## Torque Teno Virus – From Discovery to Immune Monitoring

TTV is a small, circular, non-enveloped, single-stranded DNA virus that was first described in 1997. It is characterised by high genetic diversity, and 26 species are currently classified among the genus *Alphatorquevirus* within the family Anelloviridae. To date, no causal association with any disease has been demonstrated. Its high prevalence in the general population [23, 24], replication in almost all studied body tissues and liquids [25], and high positivity rates among infants [26] have given rise to the hypothesis of TTV being a non-pathogenic, commensal virus. As such, it was attributed to the human virome [27].

Epidemiological analyses revealed higher TTV prevalences in patients with diseases causing reduced immunocompetence or chronic inflammation [10, 28]. As early as 2001, studies linked TTV prevalence to overall immunocompetence [29, 30]. In the same year, Maggi and Bendinelli speculated on the potential healthcare benefits of monitoring TTV in organ transplantation [31]. In 2003, Moen and colleagues observed steep increases in TTV load upon initiation of immunosuppression in kidney transplant recipients [32]. A decade later, associations between TTV and adverse outcomes in transplant patients became evident. In a systems biology approach, De Vlaminck and colleagues analysed the human plasma virome in patients after heart and lung transplantation, and observed not only a marked expansion of TTV after the initiation of immunosuppression but also an overall lower viral load in patients with allograft rejection [33]. Associations between TTV load and the occurrence of infections were demonstrated shortly after [34]. These findings were subsequently reproduced in a variety of different cohorts with broad consistency among studies and across different types of transplanted organs [9, 35].

Evidence for TTV as an immune marker has also emerged from studies involving non-transplant patients [36]. In patients with antineutrophil cytoplasmic antibody vasculitis, a retrospective analysis of the RAVE RCT [37] showed that those who experienced relapses had lower peripheral blood TTV loads at month 4 after therapy start, potentially reflecting insufficient immunosuppression (unpublished data from the Medical University of Innsbruck). In patients with rheumatoid arthritis, TTV loads were lower in those with persistent rheumatoid arthritis activity despite initiation of disease-modifying anti-rheumatic drug therapy, indicating an insufficient immunosuppressive effect [38].

TTV has also been proposed for triage in visits at emergency medicine outpatient clinics. Patients with a SARS-CoV-2-infection were shown to be at particularly increased risk of admission to the intensive care unit or death if TTV loads in nasopharyngeal swabs were high [39]. Promising results of TTV quantification in patients with oncologic disease were reported and recently summarised [40]. In women with ovarian cancer, those with unfavourable outcomes were found to have higher TTV loads [41]. In patients receiving chimeric antigen receptor T-cell therapy for lymphoproliferative disease, TTV dynamics were associated with therapy response and immune effector cell-associated neurotoxicity syndrome [42].

Altogether, a high TTV load has been associated with host factors and clinical conditions linked to compromised immunocompetence across heterogeneous study populations. In patients receiving immunosuppressive therapy, TTV load may be used to identify those with insufficient immune system control and increased risk of adverse outcomes. Therefore, TTV is a promising biomarker to not only quantify the net state of the immune system but also guide clinical decision-making. Ongoing and future interventional RCTs will help define the value of TTV in the clinical care of kidney transplant recipients.

## Novel Data on Torque Teno Virus Quantification Techniques

In clinical applications, TTV load is most commonly quantified in plasma; however, it can also be quantified in various body fluids, including serum, whole blood, urine, nasopharyngeal swabs and bronchoalveolar lavage. Quantifying TTV in whole blood and serum yields higher loads than in plasma. Recently, Truffot and colleagues systematically quantified this difference, analysing 216 consecutive paired samples from 68 kidney transplant recipients. They observed a mean TTV load difference of 0.4 log_10_ copies/mL (c/mL) between whole blood and plasma, with a high correlation between paired samples [43]. Unpublished data from the University of Strasbourg shows a mean TTV load difference of 0.18 log_10_ c/mL between serum and plasma among 40 solid organ transplant recipients with 169 paired samples. It is well established that viral PCRs for viruses such as cytomegalovirus (CMV) or BK polyomavirus (BKV) can show differences of up to one log between whole blood and plasma or serum, with plasma and serum providing comparable results. Future studies should investigate these relationships for TTV. Until then, we recommend quantifying TTV in plasma, as most proposed cut-offs are based on this matrix.

The most extensively reported systems for quantifying TTV load are qPCR-based and use either published primers and probes developed by Maggi and colleagues [44] or a commercially available *In Vitro* Medical Devices Regulation (IVDR)-labelled assay (TTV R-GENE^®^, bioMérieux, France) [45]. Notably, TTV loads can differ significantly between applied assays. A recent study compared the in-house PCR developed by Maggi and colleagues and the commercially available PCR in 342 samples from 314 patients, revealing a mean difference of 1.38 log_10_ c/mL (95% confidence interval: 1.30–1.46) [46]. Notably, the assays showed a high and almost linear correlation, allowing one method to be extrapolated to the other. Besides primers and probes, differences in extractors, cyclers, consumables and local standards might also lead to significant differences in TTV load quantification. Indeed, a comparison of TTV loads quantified with the same PCR assay in Fabrizio Maggi’s laboratory in Italy and at the Center for Virology at the Medical University of Vienna showed a difference of 1.0 log_10_ c/mL (unpublished data). Given these findings, it is evident that locally obtained TTV PCR results need to be cross-validated and adapted to proposed TTV load cut-offs accordingly. Such a process can be facilitated by customised quality assessment programmes like those offered by Quality Control for Molecular Diagnostics (Glasgow, UK).

For the clinical implementation of any TTV qPCR assay, a low inter- and intra-centre variability is desirable. A recent analysis by the clinical virologists of the TTVguideTX consortium demonstrated that this can be achieved for the commercial PCR using standard testing platforms [47]. In preparation for the multicentre TTVguideIT trial, the PCR was set up locally in 13 recruiting centres across Europe. Applying an internal quality control demonstrated excellent accuracy, with an inter-laboratory standard deviation of 0.19 log_10_ c/mL and an intra-laboratory standard deviation of 0.07–0.18 log_10_ c/mL. External quality assessment and linearity panels similarly showed small variability. Implementation of qPCR assays might be further supported by using automated test systems. In this regard, a study by Spezia and colleagues might be of special interest. They compared the conventional in-house PCR with an automated approach in 112 samples and found a high concordance rate [48]. Automated qPCR systems could facilitate shorter turnaround times, reduced workload and higher throughput.

## Proposed Torque Teno Virus Cut-Off Values to Guide Immunosuppression

Most data on TTV in patients receiving immunosuppressive therapy was generated in kidney transplant recipients. Findings from initial retrospective studies have since been validated by non-interventional prospective studies [49, 50]. Today, independent and robust associations have been described between low TTV loads and all types of kidney graft rejection, including clinically overt T cell-mediated rejection (TCMR), ABMR, borderline TCMR and subclinical rejection. Higher TTV loads were found prior to a broad range of infectious events, including opportunistic infections by CMV and BKV, as well as bacterial urinary tract infections. Notably, linear associations showed step-wise increasing risk constellations for both infection and rejection with increasing or decreasing TTV load, respectively, as described in a 2022 review by our group [9] and a 2023 meta-analysis by van Rijn and colleagues [51].

**FIGURE 1:**
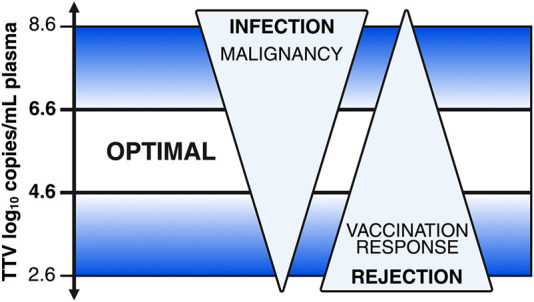
A schematic of using the host’s plasma Torque Teno virus (TTV) load for immunologic risk stratification in kidney transplant recipients. A high TTV load indicates a risk of infection and malignancy, and a low TTV load indicates a risk of rejection but associates with a better vaccination response. An optimal TTV plasma load between 4.6 and 6.6 log_10_ c/mL obtained by TTV R-GENE^®^ is proposed.

Consequently, quantifying TTV loads was proposed for risk stratification of insufficient or excessive immunosuppression in kidney transplant recipients, and the research focus shifted towards defining clinically relevant TTV load cut-offs. TTV load cut-offs that have been proposed for the care of kidney transplant recipients are presented in Table 1; Figure 1. To facilitate the interpretation of these cut-offs, we converted TTV loads obtained by in-house qPCR assays to those of the commercially available qPCR assay. Using data from prospective cohort studies, a plasma TTV load between 4.6 and 6.6 log_10_ c/mL was proposed as an optimal trade-off between the risks of rejection and infection 4–12 months after kidney transplantation [49]. Few studies have investigated TTV loads in patients beyond the first year post-transplantation. Schiemann et al. described an increased risk of rejection in patients with TTV loads <3.6 log_10_ c/mL at a median of 6 years after transplantation in a cross-sectional study (Table 2) [54]. Chauvelot and colleagues proposed a range of 3.8–5.1 log_10_ c/mL for patients between one and 4 years after transplantation and validated their findings in a prospective cohort (Table 1) [53].

**TABLE 1:** TTV load cut-offs proposed based on an IVDR-labelled qPCR assay to guide immunosuppression after kidney transplantation.

Clinical setting	Time of TTV quantification	TTV threshold
Clinically overt graft rejection^a^ (single centre, cohort) [49]	Month 4–12 post-transplant	<4.6 log_10_ c/mL^b^ ^,^ ^c^
Subclinical rejection^a^ (single centre, cohort) [52]	Month 4–12 post-transplant	<4.6 log_10_ c/mL^b^ ^,^ ^c^
Underimmunosuppression^d^ (single centre, cross-sectional) [53]	Month 12–36 post-transplant	<3.8 log_10_ c/mL^e^
Clinically overt graft rejection^a^ (single centre, cross-sectional) [54]	Median of 6 years post-transplant	<3.6 log_10_ c/mL^b^ ^,^ ^c^
Vaccine response^f^ (retrospective analysis of a multicentre RCT) [10]	Median of 7 years post-transplant	<4.6 log_10_ c/mL^b^ ^,^ ^c^
Infection^g^ (single centre, cohort) [49]	Month 4–12 post-transplant	>6.6 log_10_ c/mL^b^ ^,^ ^c^
Malignant disease^h^ (single centre cohort, unpublished)	Month 4–12 post-transplant	>6.6 log_10_ c/mL^b^ ^,^ ^c^
Overimmunosuppression^i^ (single centre, cross-sectional) [53]	Month 12–36 post-transplant	>5.1 log_10_ c/mL^e^
CMV-DNAemia >3.0 log_10_ c/mL (single centre, paediatric cohort) [55]	Year 2 (median) to year 5 (median) post-transplant	>6.3 log_10_ c/mL^b^ ^,^ ^c^
BKV-DNAemia >3.0 log_10_ c/mL (single centre, paediatric cohort) [55]	Year 2 (median) to year 5 (median) post-transplant	>5.0 log_10_ c/mL^b^ ^,^ ^c^

Abbreviations: ABMR, antibody-mediated rejection; BKV, BK polyomavirus; CMV, cytomegalovirus; c/mL, copies/mL; RCT, randomised controlled trial; SARS-CoV-2, severe acute respiratory syndrome coronavirus 2.

^a^ biopsy-proven borderline, cellular and ABMR, according to the respective Banff meeting report.

^b^ to facilitate the interpretation, TTV cut-off values obtained using an in-house PCR assay have been recalculated to match the results obtained with the TTV R-GENE^®^ qPCR assay according to the comparative study by Görzer et al. who described a mean difference of 1.38 log_10_ c/mL (95% confidence interval 1.30–1.46) between PCR methods [46].

^c^ plasma was used for TTV load quantification.

^d^ based on comparison with a healthy collective and antibody response to vaccination.

^e^ serum was used for TTV load quantification.

^f^ humoral and cellular.

^g^ defined as need for hospitalisation, anti-microbial treatment or reduction of immunosuppression.

^h^ excluding basalioma and carcinoma *in situ*.

^i^ defined by the occurrence of serious infections or malignoma.

**TABLE 2:** A selection of studies that have recently expanded the indication of TTV-guided monitoring after kidney transplantation.

Study design	Cohort	TTV monitoring	Main outcome	Main finding
**Paediatric cohort**				
Retrospective analysis of single centre cohort [55]	71 KTX included 2y after TX; 3y FUP	every 4 to 8w for 3y	10 BKV / 7 CMV infections	High TTV load associates with CMV and BKV DNAemia
**SARS-CoV-2 vaccination**				
Retrospective analysis of multicentre RCT [10]	100 KTX with mRNA vaccination; 7y after TX	At first vaccination	31 seroconversions post 2 vaccinations	High TTV load associates with impaired vaccine response
Retrospective analysis of single centre cohort [56]	459 KTX with 2 or 3 mRNA vaccination; 6y after TX	At first vaccination	208 seroconversions post 2/ 130 post 3 vaccinations	High TTV load associates with impaired vaccine response
**TTV load kinetic**				
Retrospective analysis of single centre cohort [57]	48 KTX with isolated CNI dose adjustment; 1 year after TX	0, 30, and 60d after CNI dose adjustment	TTV load	TTV load changes detectable only 2m after CNI adaption
Retrospective analysis of single centre cohort [58]	43 KTX with 5w MPA withdrawal/ 33 continued MPA; 5y after TX	0, 1m after withdrawal, 2m post reintroduction	TTV load	TTV load decreases 1m after MPA withdrawal/ increases 2m after restart
Non-randomised, open label, controlled pilot [59]	18 KTX with 2w MPA withdrawal/ 22 continued MPA; 4y after TX	0, 2w after withdrawal, 1m post reintroduction	TTV load	TTV load decreases 2 w and 1 m after MPA withdrawal
**Belatacept-based IS**				
Retrospective analysis of single centre cohort [60]	68 KTX converted from TAC to belatacept; 4y after TX	0, 3, 6, and 12m after conversion	TTV load	No significant changes of TTV load post TAC-conversion
Retrospective analysis of 2 RCTs [61]	105 KTX converted from CNI to belatacept; 2y after TX	0, 6, and 12m after conversion	TTV load	No significant changes of TTV load post CNI-conversion
**mTORi-based IS**				
Cross-sectional, single centre [54]	715 KTX, 30 mTORi-based IS; 6y after TX	at screening	TTV load	Trend towards lower TTV load in mTORi-based IS
**post-TX malignancy**				
Retrospective analysis of a single centre cohort study [50]	221 KTX; 1 year FUP	0, 7d, 1, 3, 6, and 12m	54 opportunistic infections / 11 malignancies	High TTV load associates with opportunistic infection and malignancy
Retrospective analysis of single centre cohort^a^	428 KTX; 5y FUP	Every 3m for 5y	53 malignancies 2-5y after TX	High TTV load associates with malignancy
**RCT**				
1:1: TTV-guided TAC trough level vs. SOC [11]	13 EU centres; 260 low risk KTX randomised 4m after TX; 9m FUP	Every 6w for 9m	Death, graft loss, rejection, infection	TTVguideIT: last patient last visit May 2025; results expected 2026
1:1: TTV-guided IS vs. SOC^b^	4 French centres; 300 low risk KTX randomised 1-4y after TX; 3y FUP	Every 3m for 3y	Rejection, infection, cancer, graft loss, DSA	TAOIST: first patient first visit April 2025; results expected 2030

Abbreviations: BKV, BK polyomavirus; CMV, cytomegalovirus; CNI, calcineurin inhibitor; d, days; DSA, donor-specific antibodies; DSMB, data safety monitoring board; FUP, follow-up; IS, immunosuppression; KTX, kidney transplant recipients; MPA, mycophenolic acid; m, month(s); mRNA, messenger RNA; mTORi, mammalian target of rapamycin inhibitor; TX, transplantation; RCT, randomised controlled trial; SARS-CoV-2, severe acute respiratory syndrome coronavirus 2; TAC, tacrolimus; SOC, standard of care; w, weeks y, years.

^a^ unpublished data from a retrospective analysis of the prospective TTV-POET study (DRKS ID: DRKS00012335).

^b^ study protocol published onClinicalTrials.gov(NCT06829719).

It is important to note, that validation of the proposed cut-off values guiding immunosuppressive therapy in an interventional setting is necessary prior to clinical implementation. One completed (TTVguideIT, EUCT number: 2022-500024-30-00) and one ongoing (TAOIST, ClinicalTrials.gov ID: NCT06829719) RCT will help to clarify the clinical value of TTV-guided immunosuppression in post-transplant care. In addition, these multicentre RCTs will allow for the validation of TTV load cut-offs derived from single-centre studies. TTVguideIT was a multicentre, interventional, patient- and assessor-blinded RCT conducted in 13 European centres recruiting stable adult kidney transplant recipients with low immunological risk (Table 2). The corresponding study protocol and the statistical analysis plan have been previously published [11, 62]. Between 2022 and 2024, 260 patients were randomised at month four after transplantation to either standard of care or TTV-guided tacrolimus dosing at a 1:1 ratio. TTV load was quantified in both arms every 6 weeks for 9 months. In the interventional arm, the investigators followed a protocol to set tacrolimus target trough levels according to the actual TTV load. In the control arm, TTV load was concealed. The primary endpoint was a composite of the occurrence of infections, biopsy-proven allograft rejection, graft loss and death scored by central assessors blinded to the allocation sequence. The last patient concluded the trial in May 2025.

The TAOIST trial is an interventional, open-label, parallel-group RCT being conducted in four French centres and recruiting adult kidney transplant recipients (Table 2). It aims to include 300 stable, low immunological-risk patients from months 12–48 after transplantation. The patients will be randomised to either standard of care or TTV-guided immunosuppression dosing for 36 months. TTV load will be quantified every 3 months. In the interventional arm, physicians will be free to change the dosing of immunosuppressive drugs to keep the TTV load within a predefined range. The primary outcome is a composite of *de novo* DSA, biopsy-proven rejection, infection, cancer or graft loss. The first patient was recruited in April 2025.

Notably, age, sex, and body mass index, which are associated with TTV load, do not confound or modify the association between TTV load and adverse effects related to over- and under-immunosuppression [23, 24, 63, 64]. Therefore, no adjustments to TTV cut-offs are necessary based on these characteristics. Conversely, it is plausible that patients at high risk for graft rejection - such as recipients with preformed DSA (and thus non-standard induction) or *de novo* DSA, re-transplantation, or a history of ABMR - as well as those at increased risk for infection - like older, frail recipients or individuals with comorbidities - may benefit from tailored immunosuppression. This could be reflected by higher or lower TTV cut-offs. Such a concept should be evaluated in phase three trials once the ongoing phase two studies, which recruit low-risk patients, demonstrate the safety of TTV-guided monitoring.

## Timing of Torque Teno Virus-Based Immune Monitoring

TTV load cut-offs for the guidance of immunosuppression have been proposed to be useful from month 4 post-transplant. In the first 3–4 months after kidney transplantation, TTV load is not in a steady state, and thus the definition of clinically relevant cut-off values to guide immunosuppression is difficult [34, 49]. In contrast to the well-described dynamics of TTV load after initiation of immunosuppression, TTV kinetics following dose changes of immunosuppressive drugs were unknown until recently. Two cohort studies during the COVID-19 pandemic evaluating pausing antimetabolite treatment to enhance responses to SARS-CoV-2 mRNA vaccination produced the first insights (Table 2) [58, 59]. Both studies showed a reduction in TTV load between 4 and 6 weeks after pausing antimetabolite treatment, and TTV load reached baseline values within 2 months after reinitiation of antimetabolite therapy. This evidence was recently complemented by a single-centre study by Regele et al. [57], who examined 48 kidney allograft recipients with isolated calcineurin inhibitor (CNI) dosage changes from the TTV-POET study. No significant changes in TTV load were observed 1 month after the CNI dose changes. However, a median CNI dose reduction of 33% translated to a significant decrease in TTV load 2 months after the dosage change, and a 50% increase in CNI dosage caused a trend toward higher TTV loads 2 months later (Table 2). Notably, no TTV load measurements were available after 2 months, making it impossible to analyse further changes beyond that time. Integrating the findings of these studies with the consistently described peak of TTV loads at around 3–4 months after initiating immunosuppression following transplantation, the optimal time frame for quantifying the TTV load may be assumed to be every 2–4 months. It is important to note, that assessing the effect of an immunosuppressive dose change on TTV load shortly afterward is not appropriate. Due to the time lag in TTV load changes following dose adjustments in immunosuppressive therapy, measuring TTV load should only be done approximately 2 months later.

## Torque Teno Virus Load in Belatacept- and mTOR Inhibitor-Based Immunosuppression

One question that has come up repeatedly in recent years is how TTV load cut-off values defined in patients treated with CNI-based immunosuppression can be translated to other immunosuppressive regimens. In a single-centre cross-sectional study with a limited number of patients, a higher TTV load was initially observed in those on belatacept-based (*n* = 23) compared to CNI-based immunosuppression [54]. Therefore, it was hypothesised that co-stimulation blockade might have led to more potent immunosuppression or insufficient formation of TTV-specific T-cells and thus directly influenced viral control. However, two recent studies challenged this earlier finding and did not show significant increases in TTV loads after conversion from CNIs to belatacept (Table 2) [60, 61, 65]. In a retrospective study in Grenoble that included 68 patients converted from CNI to belatacept at a median of 4 years after transplantation, TTV loads did not change significantly from baseline [60]. A retrospective analysis of two RCTs examined TTV loads in 105 patients randomised to either CNI continuation or conversion to belatacept at 6 and 12 months after conversion [61]. Those who switched to a belatacept-based regimen showed stable TTV loads and no significant differences in TTV dynamics compared to those who maintained CNI-based therapy at both time points. In both studies, while infection and rejection rates were low, precluding any meaningful analysis, TTV loads tended to be higher in patients with subsequent infections and lower before rejection episodes. These data suggest the potential application of TTV cut-off values defined using CNI-based regimens for risk stratification also in patients on belatacept-based immunosuppression.

Unlike belatacept-based immunosuppression, insufficient data is available for mammalian target of rapamycin inhibitor (mTORi)-based immunosuppression to make recommendations concerning the clinical value of TTV-guided immunosuppression. In only one retrospective cross-sectional single-centre study, the 30 patients receiving mTORi-based immunosuppression showed absolute but not statistically significant lower TTV loads than those receiving CNI-based immunosuppression (Table 2) [54].

## Torque Teno Virus Monitoring in Paediatric Kidney Transplant Recipients

Until recently, cut-off values for TTV-guided immunosuppression were unavailable for paediatric kidney transplant recipients, and data were mainly derived from one cross-sectional study and one single-centre cohort study associating Anelloviridae trajectories with immunosuppression and graft rejection [66, 67]. Recently, a larger retrospective single-centre study by Eibensteiner and colleagues added to these data (Table 2). They retrospectively analysed all paediatric kidney transplant recipients followed between 2014 and 2020 at the Medical University of Vienna for TTV load at 4–8 weeks intervals in the context of CMV and BKV infections (*n* = 71) [55]. They described a higher TTV load in patients with subsequent CMV and BKV infections during a 3-year follow-up and defined cut-offs at 7.7 and 6.4 log_10_ c/mL, respectively, to predict subsequent clinically relevant viral DNAemia (Table 2). Recipients in the cohort showed a wide age range (IQR 3.5–13.2 years at transplantation), and it is well established that TTV load increases with age [24, 68]. Analyses from adult kidney transplant cohorts have demonstrated an association between TTV load and adverse effects related to both over- and under-immunosuppression across all age groups [49]. Future research should explore whether this relationship also applies to the full paediatric recipient population.

## Novel Endpoints for Torque Teno Virus-Based Monitoring: Malignancy and Vaccination Response

The consequences of intense immunosuppression go beyond an elevated incidence of infections, as such patients are also at an increased risk of oncologic disease. Until recently, only one single-centre cohort study that included 221 patients indicated higher TTV loads in kidney transplant recipients with subsequent oncologic disease. Notably, oncologic disease was analysed only within a combined endpoint that included mainly opportunistic infections (Table 2) [50]. An yet unpublished analysis of data from the prospective TTV-POET study (DRKS ID: DRKS00012335) showed that the cumulative TTV load in 428 patients from months 4–12 after transplantation was predictive for the development of malignoma (53 events) in the subsequent 4 years of follow-up. Using the Vienna in-house PCR, patients with a TTV load >8 log_10_ c/mL had a significantly higher risk of developing cancer than those with a TTV load <8 log_10_ c/mL. Notably, this TTV load cut-off is equivalent to the cut-off for defining patients at risk of infection (Table 1). Therefore, it can be speculated that targeting the optimal TTV load range proposed to reduce infection might also reduce malignancy rates.

Another clinically relevant side effect of immunosuppression in kidney transplant recipients is a reduced response to vaccination, and recent studies have analysed TTV loads in this context. Graninger and colleagues retrospectively analysed 100 kidney transplant recipients from samples prospectively stored by an interventional German multicentre RCT, demonstrating that TTV loads were lower in serological responders than in non-responders after two doses of the SARS-CoV-2 mRNA vaccine. Those with TTV loads >10^6^ c/mL showed no cellular immune response, and only 12% showed a serological response (Table 2) [10]. These findings were confirmed by a retrospective single-centre study involving 459 kidney transplant recipients receiving their second dose of the SARS-CoV-2 mRNA vaccine, of which half then received a third dose (Table 2) [56]. Notably, during the COVID-19 pandemic, temporal antimetabolite withdrawal was shown to enhance responses to SARS-CoV-2 vaccination in some [58, 69] but not all [59] RCTs. Therefore, a TTV-based approach to individualise both the amount and timing of reducing immunosuppression before planned vaccination could be an interesting design to evaluate in an interventional trial.

## Scenarios Where Torque Teno Virus Load Might Not Reflect Immune Function Accurately

Besides insufficient data on patients receiving mTORi-based immunosuppression, as mentioned above, there are other scenarios where TTV-guided immunosuppression might be challenging. In a few patients, the TTV load does not reach the detection limit of the commonly used qPCRs. In these cases, differentiating between non-infection and DNAemia below the detection limit is difficult, and the TTV load cannot be used for risk stratification of immunosuppression. Quantification of other genera of the Anelloviridae family might help in this situation. Recently, a study involving 168 solid organ transplant recipients showed that quantifying *Betatorquevirus* (formerly the Torque Teno Mini virus) and *Gammatorquevirus* (formerly the Torque Teno Midi virus) improved the prediction of the SARS-CoV-2 vaccination response [70].

TTV was shown to become almost undetectable following myeloablative conditioning regimens in hematopoietic stem cell transplantation patients [71, 72]. Similarly, TTV load significantly decreases during anti-thymocyte globulin treatment in solid organ transplant recipients [73]. In both settings, the TTV load cannot be used to quantify immunosuppression. Notably, TTV loads returned to baseline 1–2 weeks after anti-thymocyte globulin treatment and might reflect immunosuppression accurately again after that time point [73]. In concordance with the above mentioned findings leukocytes were proposed as a replication pool for TTV and it may be speculated upon whether the validity of TTV loads is also reduced during episodes of significant leukopenia due to, for example, CMV infection or drug toxicity.

## Summary and Outlook

The association between TTV load and adverse outcomes linked to immune function in kidney transplant recipients is increasingly robust, and cut-offs for TTV-guided immunosuppression have been proposed. Recent studies involving paediatric cohorts, patients with belatacept-based immunosuppression and endpoints other than infection and rejection might broaden its potential clinical applications. Future studies must focus on knowledge gaps, including TTV loads in patients at later time points after transplantation and recipients on mTORi-based immunosuppression. Improved quantification methods of TTV load have been proposed to support implementation, which will depend on the results of ongoing interventional trials. Following results of ongoing interventional trials, TTV will have to position itself in the context of other emerging biomarkers for immunologic monitoring.
